# Association of lactate dehydrogenase and diabetic retinopathy in US adults with diabetes mellitus

**DOI:** 10.1111/1753-0407.13476

**Published:** 2023-09-25

**Authors:** Ping Yang, Weiwei Xu, Ling Liu, Gangyi Yang

**Affiliations:** ^1^ Department of Critical Care Medicine Second Affiliated Hospital of Chongqing Medical University Chongqing China; ^2^ Department of Endocrinology and Metabolism Second Affiliated Hospital of Chongqing Medical University Chongqing China; ^3^ Department of Ophthalmology Chongqing University Central Hospital, Chongqing Emergency Medical Center Chongqing China

**Keywords:** diabetic retinopathy, lactate dehydrogenase, NHANES, type 2 diabetes mellitus

## Abstract

**Objectives:**

The purpose of our investigation is to evaluate the level of relationship between lactate dehydrogenase (LDH) and the occurrence of diabetic retinopathy (DR) in adults with diabetes mellitus (DM).

**Methods:**

The investigation involved an analysis of five sectional data cycles acquired from the National Health and Nutrition Examination Survey from 2009 to 2018. The present study involved the selection of DM samples from a complex multistage probability sample. These samples were subsequently classified into two distinct groups, namely the No DR (NDR) and DR groups. The present study comprehensively investigated the biological and social risk factors associated with DR. The biological factors examined in this investigation included blood pressure, blood routine, hemoglobin A1c, blood glucose, and comorbidities. The social dimensions encompass education and sex.

**Results:**

After considering all factors, multivariate regression models indicated a significant relationship between DR and increased LDH (adjusted odds ratio = 1.007, 95% confidence interval: 1.003–1.011). The subgroup analysis revealed that the effect size of LDH on the existence of DR in the subgroups remained consistent, as indicated by all *p* values greater than .05. A statistically significant relationship was identified between elevated LDH levels > 134 U/L and a raised risk of DR in people with DM.

**Conclusion:**

LDH concentrations were connected with an increased prevalence of DR in participants with DM. Our study highlights that patients with LDH > 134 U/L are distinguishably related to DM complicated by DR. DR is more common in diabetic individuals with coronary heart disease.

## Graphical abstract

- There are limited studies to reveal the association between lactate dehydrogenase (LDH) and diabetic retinopathy (DR). Our findings were the initial ones to demonstrate a positive correlation between the rise in LDH levels and the prevalence of DR. Our study revealed that diabetic individuals exhibiting LDH levels > 134 U/L were found to have a statistically significant elevation in their probability of developing DR. After adjusting for other confounding factors, there was a relationship observed between LDH and the incidence of DR. Furthermore, our research indicates that individuals diagnosed with diabetes and coronary heart disease exhibit heightened susceptibility to DR.

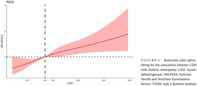

## 1 INTRODUCTION

Recently, the prevalence of diabetes has been growing annually. Some studies have estimated that by 2040, the proportion of adult diabetics worldwide is expected to increase to 10.4%, or about 642 million diabetics.^1^ Diabetes mellitus (DM) presents a significant risk to human health, with diabetic retinopathy (DR) being a dangerous complication and the leading cause of vision impairment in individuals with DM.^2^, ^3^, ^4^, ^5^

The principal pathophysiological mechanism underlying DR involves a variety of alterations resulting from hyperglycemia, such as the thickening of basement membrane of retinal capillaries, increased permeability of retinal vasculature, tissue ischemia, and the generation of various vasoactive agents, resulting in the development of new blood vessels.^6^, ^7^, ^8^, ^9^ Oxidative stress (OS) is essential in the pathophysiology of DR. The overabundance of reactive oxygen species has the potential to cause damage to tissues located in the surrounding area of retinal blood vessels, ultimately resulting in the development of DR.^6^, ^10^, ^11^

Lactate dehydrogenase (LDH) is a glycolytic enzyme that significantly affects metabolic OS. The amount of released LDH is minimal, but it increases significantly when the permeability of the cell membrane enhances. The cumulative amount of LDH released positively correlates with the number of damaged cells. Research indicated that unstable hyperglycemia can stimulate the action of protein kinase C and trigger an OS response. Elevated glucose levels and lack of oxygen have been observed to increase LDH levels in retina cells.^12^

Several animal trials have revealed the contribution of OS to the advancement of DR.^13^, ^14^, ^15^ Some researchers indicated that LDH could be used as a biochemical parameter of OS to predict the occurrence of DR.^13^, ^16^, ^17^ Yu‐Shan Hsieh et al demonstrated that raised LDH is connected with glycated albumin (ALB) and insulin antibody levels.^18^ Ye‐Li Wang et al did not observe the association between LDH and DM risk in Singapore Chinese.^19^ Nonetheless, the relationship between LDH and DM or DR is unclear. Furthermore, limited studies have revealed the association between LDH and DR. Consequently, a cross‐sectional study was performed to test the association between LDH and DR.

## 2 MATERIALS AND METHODS

### 2.1 Study design and participant recruitment

**FIGURE 1:**
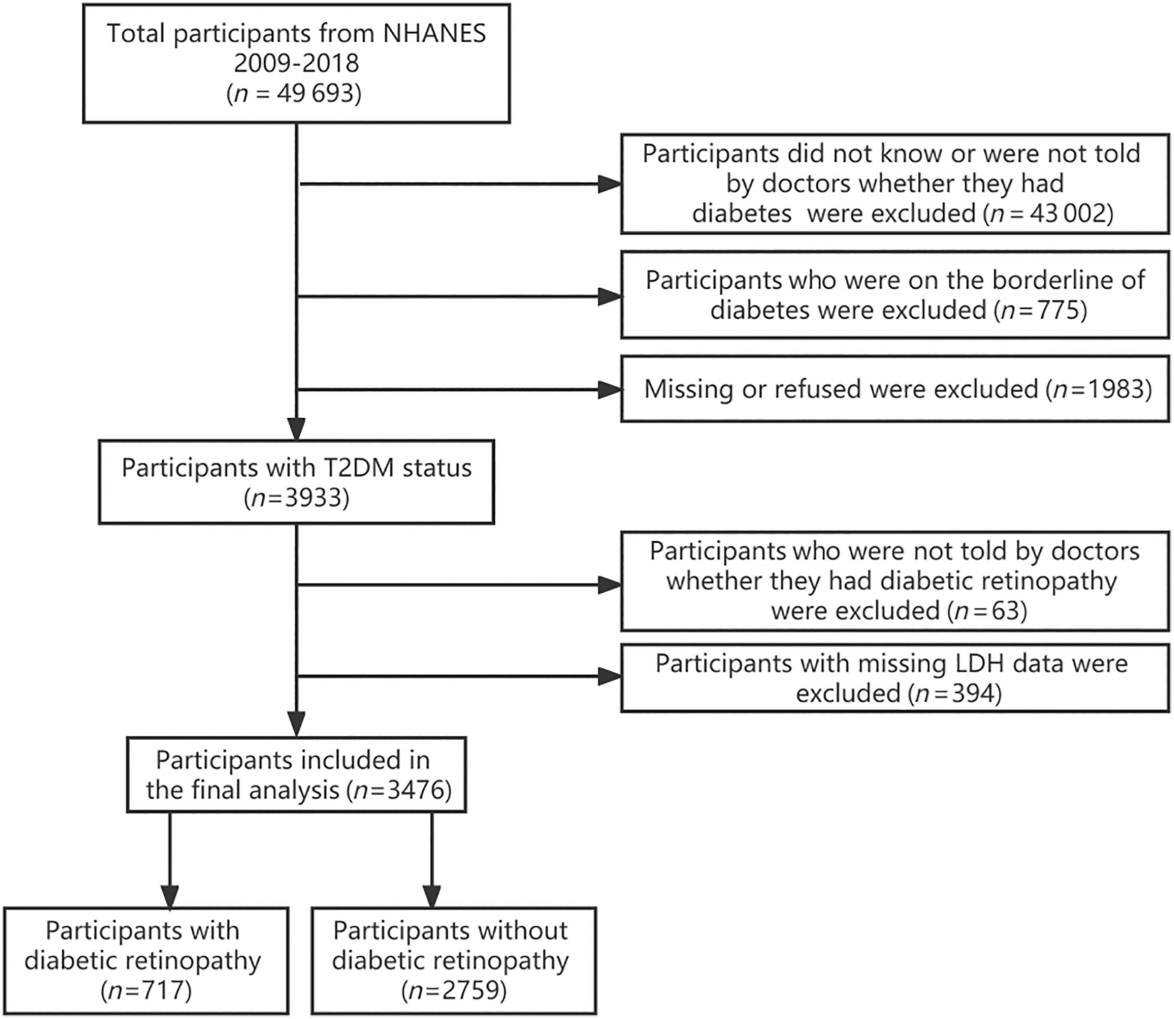
Flow chart of the selection process. ALB, albumin; ALP, alkaline phosphatase; ALT, alanine aminotransferase; BUN, blood urea nitrogen; CHD, coronary atherosclerotic heart disease; CR, creatinine; FGLU, fasting glucose; HbA1C, hemoglobin A1C; LYM, lymphocyte number; MON, monocyte number; OR, odds ratio; UA, uric acid; WBC, white blood cell count.

The information utilized in this investigation was acquired from the National Health and Nutrition Examination Survey (NHANES) spanning from 2009 to 2018. This study enrolled a cohort of 3933 participants who met the eligibility principles for having DM status. According to the flow chart depicted in Figure 1, individuals with incomplete LDH assessment data and participants who did not receive confirmation from medical professionals regarding their DR status were excluded. The National Center for Health Statistics institutional review board accepted the investigation ethics, and the study design was established according to the Declaration of Helsinki. Before enrollment, all participant gave their informed consent.

### 2.2 Exposure

The serum LDH investigation was made available to individuals aged 20 years and above during the NHANES study conducted between 2009 and 2018. The Laboratory/Medical Technologists Procedure Manual provides comprehensive guidance on collecting and processing samples in NHANES. The vials are maintained at suitable freezing conditions of −30°C until they are dispatched to the National Center for Environmental Health for analysis.

### 2.3 Definition of DM and DR

From NHANES 2009 to 2018, we identified from the questionnaire data patients with DM and DR who were informed by doctors.

### 2.4 Covariates

The covariates considered in this study were sex, age, race/ethnicity, body mass index (BMI), and education. Education was categorized into four groups, namely primary school, secondary school, college, or above, and not recorded. Race was separated into five groups, Mexican American, non‐Hispanic White, other Hispanic, non‐Hispanic Black, and others. Laboratory data such as total bilirubin, albumin (ALB), alanine aminotransferase (ALT), gamma glutamyl transferase, aspartate aminotransferase, blood urea nitrogen (BUN), alkaline phosphatase (ALP), creatinine, cholesterol, triglycerides (TRG), uric acid, iron, sodium, potassium, calcium, phosphorus, white blood cell count (WBC), lymphocyte number (LYM), red blood cells (RBC), monocyte number (MON), neutrophils number, eosinophils number (EON), platelet count (PLT), hemoglobin, hematocrit (HCT), red cell distribution width, hemoglobin a1c (HbA1C), fasting glucose (FGLU), and low‐density lipoprotein‐cholesterol (LDL) were detected by standard procedures. We also included chronic disease covariates, hypertension (HBP) and coronary atherosclerotic heart disease (CHD), to disentangle the association between LDH and DR.

### 2.5 The management of missing data

For continuous variables (BMI, calcium, iron, WBC, LYM, MON, neutrophils number, EON, RBC, hemoglobin, HCT, red cell distribution width, PLT, and HbA1C) with a lesser amount of missing values (< 100), mean interpolation is used. Continuous variables (FGLU, TRG, LDL) with a larger quantity of missing data (> 100) are transformed into categorical variables, with the missing values being grouped separately, and the dummy variable method is applied for interpolation. The missing variables are isolated into a separate group for categorical variables (HBP, CHD) with missing data, and the dummy variable method is used for interpolation.

### 2.6 Statistical analysis

The analytical procedures used sample weights, stratification, and clustering to address complicated sampling designs following the NHANES analytical directions. This investigation conducted all analyses using the total sample 2‐year Mobile Examination Center examination weight (WTMEC2YR) obtained from the NHANES database. Descriptive statistics for the continuous and categorical variables were reported in the form of means, SDs, and frequencies, respectively. The *t* test was employed to make comparisons between continuous variables. The chi‐square statistical test was employed to assess the variations among categorical variables. The study employed logistic regression models to estimate 95% confidence intervals (CIs), and the odds ratios (ORs) to identify the association between LDH and DR. Model 1 was unadjusted.

In contrast, model 2 was adjusted for age, sex, and race. Model 3 was adjusted for age, sex, race, education, ALB, ALT, ALP, BUN, creatinine, uric acid, WBC, LYM, MON, HbA1C, FGLU, and CHD. The logistic regression model's interaction test was used to contrast the ORs among the examined subgroups.

The study employed the restricted cubic sinus regression technique to model the relationship between LDH and DR. The study used the median of LDH as the reference values and placed knots at the 25th, 50th, 75th, and 95th percentiles.

The statistics packages R (version 4.2.0) from The R Foundation (http://www.r-project.org), EmpowerStats from X&Y Solutions, Inc. in Boston, Massachusetts (www.empowerstats.net), as well as SPSS 26.0 and Stata 17.0, were used for data analysis.

## 3 RESULTS

### 3.1 Participants characteristics

Tables 1 and 2 indicate the medical and demographic characteristics of 3476 people. The results of this investigation did not show statistical variations in age, BMI, race, aspartate aminotransferase, cholesterol, gamma glutamyl transferase, iron, uric acid, EON, red cell distribution width, PLT, TRG, LDL, and HBP. Among the participants, the ALP, BUN, creatinine, LDH, potassium, HbA1C, and FGLU of the patients with DR were higher (*p* < .05). Patients with DR are more likely to have CHD (*p* < .05), and with the improvement in education level, the prevalence of DR gradually decreased (*p* < .05). Patients with DR had low ALB, ALT, calcium, total bilirubin, sodium, WBC, LYM, MON, RBC, hemoglobin, and HCT (*p* < .05).

**TABLE 1:** Characteristics of participants with or without diabetic retinopathy.

Characteristic	Total	NDR	DR	*p* value
	(*n* = 3476)	(*n* = 2759)	(*n* = 717)	
Age (years)	59.83 ± 13.75	59.80 ± 13.83	59.98 ± 13.41	.77
Sex (%)				.45
Male	52.65	52.95	51.31	
Female	47.35	47.05	48.69	
Race (%)				.31
Mexican American	9.78	9.76	9.91	
Other Hispanic	5.63	5.37	6.79	
Non‐Hispanic White	61.07	61.81	57.79	
Non‐Hispanic Black	13.88	13.70	14.67	
Other race	9.64	9.37	10.84	
Education (%)				<.01
Primary school	9.51	9.02	11.66	
Secondary school	36.74	35.43	42.50	
College or above	52.88	54.52	45.66	
Not recorded	0.87	1.03	0.18	
HBP (%)				.69
Yes	96.13	96.07	96.37	
No	3.83	3.90	3.51	
Not recorded	0.05	0.03	0.12	
CHD (%)				<.01
Yes	12.57	11.62	16.74	
No	86.82	87.75	82.72	
Not recorded	0.61	0.63	0.54	
BMI (kg/m^2^)	33.03 ± 7.56	33.02 ± 7.50	33.05 ± 7.84	.94

Abbreviations: BMI, body mass index; CHD, coronary atherosclerotic heart disease; DR, diabetic retinopathy; HBP, hypertension; NDR, no diabetic retinopathy.

**TABLE 2:** Characteristics of participants with or without diabetic retinopathy.

Characteristic	Total	NDR	DR	*p* value
	(*n* = 3476)	(*n* = 2759)	(*n* = 717)	
ALB (g/L)	41.12 ± 3.54	41.29 ± 3.48	40.34 ± 3.68	<.01
ALT (U/L)	26.09 ± 28.81	26.57 ± 31.15	23.97 ± 14.42	.04
AST (U/L)	25.82 ± 20.47	26.14 ± 21.88	24.42 ± 12.38	.05
ALP (U/L)	76.09 ± 32.74	75.37 ± 33.76	79.29 ± 27.57	<.01
BUN (mmol/L)	6.04 ± 2.89	5.85 ± 2.62	6.87 ± 3.77	<.01
CA (mmol/L)	2.35 ± 0.10	2.36 ± 0.10	2.33 ± 0.11	<.01
CHO (mmol/L)	4.59 ± 1.193	4.60 ± 1.19	4.55 ± 1.22	.33
CR (umol/L)	89.47 ± 64.01	85.36 ± 49.96	107.58 ± 103.62	<.01
GGT (U/L)	34.27 ± 46.49	34.52 ± 48.57	33.19 ± 35.96	.51
FE (umol/L)	13.91 ± 5.59	13.94 ± 5.54	13.78 ± 5.81	.52
LDH (U/L)	138.74 ± 36.39	136.80 ± 32.64	147.23 ± 48.71	<.01
TB (umol/L)	10.08 ± 4.95	10.18 ± 5.07	9.67 ± 4.34	.02
Uric acid (umol/L)	338.37 ± 92.33	338.57 ± 90.25	337.51 ± 100.96	.79
NA (mmol/L)	138.99 ± 2.90	139.05 ± 2.85	138.71 ± 3.07	<.01
K (mmol/L)	4.12 ± 0.41	4.10 ± 0.39	4.18 ± 0.45	<.01
WBC (10^3^/UL)	7.82 ± 2.24	7.89 ± 2.27	7.53 ± 2.11	<.01
LYM (10^3^/UL)	2.150 ± 0.93	2.18 ± 0.97	2.00 ± 0.75	<.01
MON (10^3^/UL)	0.60 ± 0.20	0.60 ± 0.21	0.57 ± 0.19	<.01
NEN (10^3^/UL)	4.80 ± 1.75	4.83 ± 1.76	4.68 ± 1.68	.06
EON (10^3^/UL)	0.23 ± 0.18	0.23 ± 0.18	0.23 ± 0.16	.61
RBC (10^6^/UL)	4.631 ± 0.52	4.66 ± 0.51	4.52 ± 0.57	<.01
HB (g/dL)	13.87 ± 1.59	13.94 ± 1.58	13.53 ± 1.58	<.01
HCT (%)	41.13 ± 4.44	41.34 ± 4.40	40.22 ± 4.49	<.01
RDW (%)	13.77 ± 1.38	13.77 ± 1.38	13.76 ± 1.35	.79
PLT (10^3^/UL)	235.12 ± 70.76	235.80 ± 71.00	232.14 ± 69.64	.24
HbA1C (%)	7.34 ± 1.68	7.25 ± 1.65	7.77 ± 1.75	<.01
FGLU (mmol/L)	8.85 ± 3.46	8.71 ± 3.33	9.52 ± 3.91	<.01
TRG (mmol/L)	1.78 ± 1.75	1.80 ± 1.83	1.64 ± 1.26	.15
LDL (mmol/L)	2.54 ± 0.93	2.54 ± 0.91	2.53 ± 1.04	.85

*Note*: Mean +/− SD for age, ALB, ALT, AST, BUN, CHO, CR, GGT, LDH, TB, uric acid, NA, K, BMI, ALP, CA, FE, WBC, LYM, MON, NEN, EON, RBC, HB, HCT, RDW, PLT, and HbA1C. The *p* value was determined using a weighted linear regression model. % for sex, race, education, FGLU, TRG, LDL, HBP, CHD. The determination of *p* value was performed employing a weighted chi‐square test.

Abbreviations: ALB, albumin; ALP, alkaline phosphatase; ALT, alanine aminotransferase; AST, aspartate aminotransferase; BMI, body mass index; BUN, blood urea nitrogen; CA, calcium; CHD, coronary atherosclerotic heart disease； CHO, cholesterol; CR, creatinine; DR, diabetic retinopathy; EON, eosinophils number; FE, iron; FGLU, fasting glucose; GGT, gamma‐glutamyl transferase; HB, hemoglobin; HbA1C, hemoglobin A1c; HBP, hypertension; HCT, hematocrit; K, potassium; LDH, lactate dehydrogenase; LDL, Low‐density lipoprotein‐cholesterol; LYM, lymphocyte number; MON, monocyte number; NA, sodium; NDR, no diabetic retinopathy; NEN, neutrophils number; PLT, platelet count; RBC, red blood cell; RDW, red cell distribution width; TB, total bilirubin; TRG, triglyceride; WBC, white blood cell count.

### 3.2 Association between lactate dehydrogenase and diabetic retinopathy

After adjusting for possible confounders, the OR for LDH and DR was 1.007 (95% CI, 1.003–1.011). Compared to the highest quartile of LDH (≥156 U/L) and the lowest quartile (≤116 U/L), the OR was 2.033 (Table 3).

**TABLE 3:** Relationship between LDH and DR.

		Model 1			Model 2			Model 3		
		OR	95% CI	*P* value	OR	95% CI	*p* value	OR	95% CI	*p* value
LDH (U/L)		1.008	(1.004，1.011)	<.001	1.008	(1.004，1.011)	<.001	1.007	(1.002，1.011)	.002
LDH quartile (U/L)	No.									
Q1 (≤116)	838	1 (reference)			1 (reference)			1 (reference)		
Q2 (117–134)	878	1.476	(1.052，2.070)	.024	1.493	(1.063，2.097)	.021	1.526	(1.078，2.160)	.017
Q3 (135–155)	863	1.644	(1.174，2.301)	.004	1.667	(1.188，2.341)	.003	1.549	(1.089，2.204)	.015
Q4 (≥156)	897	2.253	(1.593，3.186)	<.001	2.293	(1.605，3.276)	<.001	2.105	(1.395，3.177)	<.001
*p* for trend		<.001			<.001			<.001		

Abbreviations: CI, confidence interval; DR, diabetic retinopathy; LDH, lactate dehydrogenase; OR, odds ratio.

*Note*: Model 1 unadjusted. Model 2 adjusted for age, sex, and race. Model 3 adjusted for age, sex, race, education, albumin, alanine aminotransferase, alkaline phosphatase, blood urea nitrogen, creatinine, hemoglobin A1c, uric acid, white blood cell count, lymphocyte number, monocyte number, fasting glucose, coronary atherosclerotic heart disease.

**FIGURE 2:**
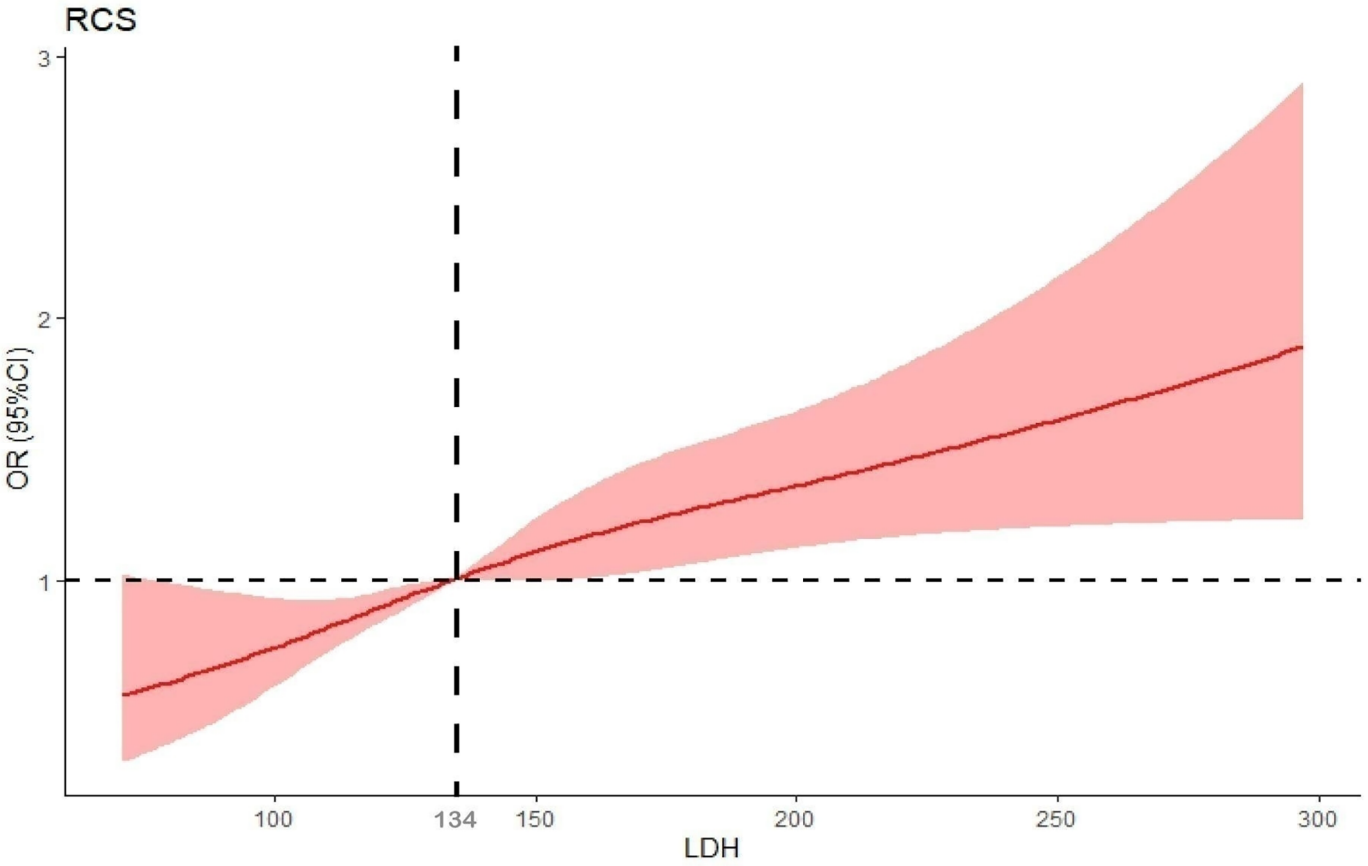
Restricted cubic spline fitting for the association between LDH with diabetic retinopathy. LDH, lactate dehydrogenase; NHANES, National Health and Nutrition Examination Survey; T2DM, type 2 diabetes mellitus.

The present study used the restricted cubic spline (RCS) method (Figure 2) and established the median LDH value of 134 U/L as the reference point. Our findings revealed a nonlinear association between the continuously measured LDH levels and DR, with a statistically significant *p* value < .001. ORs relating to the correlation between LDH and DR exhibited an increase in the presence of raised LDH levels. A statistically significant association was observed between LDH levels > 134 U/L and an OR > 1.00.

**FIGURE 3:**
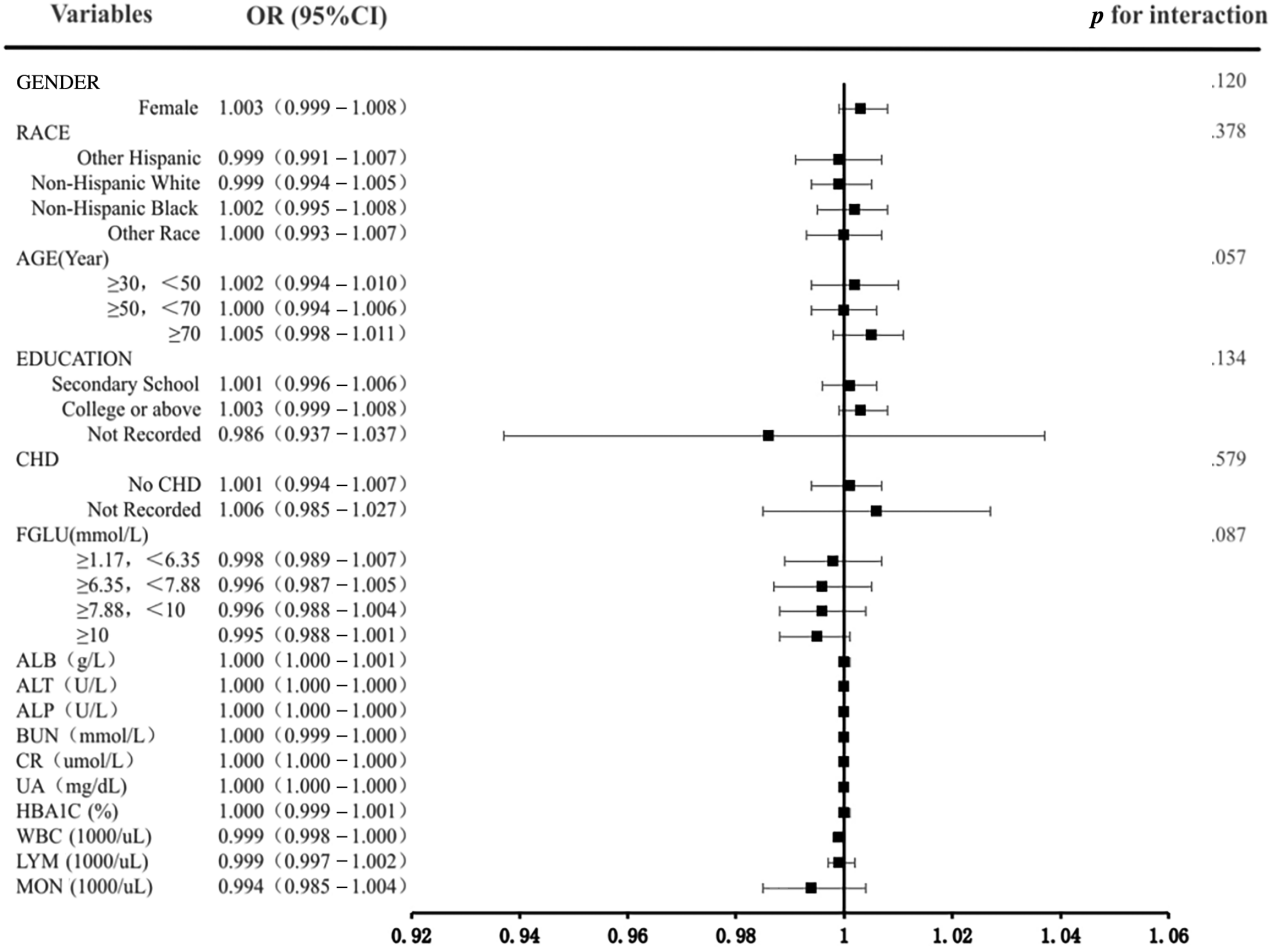
Odds of DR subgroups based on the LDH with various clinical and laboratory variables. DR, diabetic retinopathy; LDH, lactate dehydrogenase; OR, odds ratio; RCS, restricted cubic spline.

Subgroup examinations were conducted to detect the relationship robustness between LDH and DR. In the adjusted model 3, there were no significant interaction effects between subgroup variables and LDH (*p* interaction > .05). The results exhibited that the association of LDH was comparable in most subpopulations (Figure 3).

## 4 DISCUSSION

As far as our understanding goes, our findings were the initial ones to demonstrate a positive correlation between the increase in LDH levels and the prevalence of DR. Our study revealed that diabetics who have LDH levels > 134 U/L were found to have a statistically significant increase in their probability of developing DR. After adjusting for other confounding factors, there was a relationship observed between LDH and the incidence of DR. Furthermore, our research indicates that individuals diagnosed with diabetes and CHD exhibit greater susceptibility to DR.

Several investigations revealed that individuals with type 2 DM with higher HbA1c values are at elevated risk of developing DR compared to those with lower HbA1c values.^20^, ^21^ Our investigation findings showed that the level of HbA1c in the DR group was significantly greater than that detected in the NDR group (*p* < .01). Because HbA1C is one of the diagnostic criteria for diabetes, it is challenging to use it as a single indicator to predict whether patients have a risk of DR. Hongyan Yang et al discovered that raised LDH was also a risk factor for DR.^22^ Our investigation also confirmed the association between LDH and DR. Moreover, modeling and interaction research eliminated the interference of other confounding factors in the research results.

Our study demonstrated no significant variation in uric acid concentrations between the DR and NDR groups. There is still no clear conclusion on the association between DR and uric acid. Chen et al established a relationship between the severity of DR in individuals with DM and the levels of uric acid and urinary albumin.^23^ Qun Xia et al discovered that individuals with DM with elevated uric acid concentrations are associated with a higher prevalence of diabetic nephropathy but not DR.^24^ Guo Yi Cong et al confirmed that DR patients had higher uric acid levels than the control group, but the alteration was statistically nonsignificant at the early stage. Instead of forecasting the development of DR, uric acid may be a possible marker for determining disease severity in diabetic patients with DR.^25^

Our research also found that the WBC, LYM, and MON of the NDR group were substantially greater than those of the DR group. This is inconsistent with most previous research findings.^26^, ^27^ This is due to the low sensitivity and poor stability of neutrophils, monocytes, and lymphocytes to various physiological and pathological conditions. Therefore, currently in clinical practice, platelet‐to‐lymphocyte ratio and neutrophil‐to‐lymphocyte ratio are chosen as possible markers to reflect the status of inflammatory and immune response status.^28^

We detected no significant variations in HBP between diabetics with DR and those without. HBP was a high‐risk factor for DR, according to the UK Prospective Diabetes Study.^29^, ^30^ Most previous cross‐sectional studies^31^, ^32^ did not elucidate the causal relationship between pulse pressure, systolic blood pressure, and DR. The results of a meta‐analysis involving a considerable sample size of 10 229 individuals indicated a significant correlation between altered retinal blood vessels and an increased likelihood of HBP. This association persisted for up to 10 years following the initial change. This observation supports the hypothesis that alterations in retinal microcirculation may occur before the onset of arterial HBP.^33^, ^34^ When reviewing our cross‐sectional study, we did not mention the duration of HBP in individuals with DM, and we did not find an association between HBP and DR.

Our research discovered that patients with diabetes with CHD are more likely to have DR. Among the Hisayama cohort study subjected to participants in the screening examination in 1988, the coronary atherosclerotic grade, pathologically estimated in luminal stenosis or incidence of advanced lesions of coronary arteries or both, was significantly correlated with serum HbA1c levels. These outcomes also revealed that impaired glucose metabolism is a substantial risk factor for coronary atherosclerosis.^35^ In diabetic patients, microvascular lesions caused by inflammation and OS accumulate in the coronary arteries and form coronary atherosclerosis. DR is also the result of diabetic blood vessel damage. Therefore, there is a clear association between DR and CHD.

The investigation has a few restrictions. First, the NHANES study did not include an oversampling of Asian Americans or individuals of races or ethnicities other than the White, Black, and Hispanic categories. As a result, the data obtained may not be widely applicable to the mentioned groups. To address this limitation, a multicenter investigation may be conducted to confirm the findings. Second, the diagnosis of diabetic retinopathy in the five cycles of study was solely based on a questionnaire survey, lacking the results of retinal imaging examination, which makes it difficult to determine the accuracy of information. Third, the use of a cross‐sectional design in this study prevented the establishment of a temporal connection between LDH and DR, thus limiting our ability to determine causal relationships. Therefore, further investigation using longitudinal data is necessary to delve deeper into this topic. Fourth, the inherent lack of data may introduce selection bias, and although interpolation techniques can be employed to address this concern, they may potentially affect the research findings. Moreover, LDH is a nonspecific marker that can be elevated in acute and chronic conditions. As mentioned earlier, this study cannot establish the causal relationship between LDH and DR, so further research is required to prove whether LDH is a predictive factor for DR.

## 5 CONCLUSION

We discovered that a raised LDH level was connected to an elevated incidence of DR. Our research found that when LDH is > 134 U/L, LDH and DR are positively correlated. In our subsequent research, we plan to conduct a cohort study to investigate the potential of LDH as a predictive factor for DR. At the same time, diabetic patients with CHD should be advised to strengthen DR screening to prevent disability.

## AUTHOR CONTRIBUTIONS

Gangyi Yang: contributed to the conception and design, the drafting, and revision of the article. Ling Liu: contributed to the conception and design, the reviewing of the article, or critical revision of important intellectual content. Materials preparation and data analysis were performed by Ping Yang. Weiwei Xu: collected data and wrote the draft. All authors approved the final version and agreed to be responsible for all aspects of the work.

## FUNDING INFORMATION

The study did not receive any external financial support.

## DISCLOSURE

The authors declare that they have no conflict of interest.

## Data Availability

Data sets generated and analyzed during the current study are available on the NHANES website, https://www.cdc.gov/nchs/nhanes/index.htm.

## References

[1] Ogurtsova K , da Rocha Fernandes JD , Huang Y , et al. IDF diabetes atlas: global estimates for the prevalence of diabetes for 2015 and 2040. Diabetes Res Clin Pract. 2017;128:40‐50. doi:10.1016/j.diabres.2017.03.024 28437734

[2] Bourne RR , Stevens GA , White RA , et al. Causes of vision loss worldwide, 1990‐2010: a systematic analysis. Lancet Glob Health. 2013;1(6):e339‐e349. doi:10.1016/s2214-109x(13)70113-x 25104599

[3] Nentwich MM , Ulbig MW . Diabetic retinopathy–ocular complications of diabetes mellitus. World J Diabetes. 2015;6(3):489‐499. doi:10.4239/wjd.v6.i3.489 25897358 PMC4398904

[4] Heintz E , Wiréhn AB , Peebo BB , Rosenqvist U , Levin LA . Prevalence and healthcare costs of diabetic retinopathy: a population‐based register study in Sweden. Diabetologia. 2010;53(10):2147‐2154. doi:10.1007/s00125-010-1836-3 20596693

[5] Chen YY , Chen YJ . Association between dietary calcium and potassium and diabetic retinopathy: a cross‐sectional retrospective study. Nutrients. 2022;14(5):1086. doi:10.3390/nu14051086 35268061 PMC8912727

[6] Kang Q , Yang C . Oxidative stress and diabetic retinopathy: molecular mechanisms, pathogenetic role and therapeutic implications. Redox Biol. 2020;37:101799. doi:10.1016/j.redox.2020.101799 33248932 PMC7767789

[7] Rodríguez ML , Pérez S , Mena‐Mollá S , Desco MC , Ortega ÁL . Oxidative stress and microvascular alterations in diabetic retinopathy: future therapies. Oxid Med Cell Longev. 2019;2019:4940825. doi:10.1155/2019/4940825 31814880 PMC6878793

[8] Andersen N , Hjortdal J , Schielke KC , et al. The Danish registry of diabetic retinopathy. Clin Epidemiol. 2016;8:613‐619. doi:10.2147/clep.S99507 27822108 PMC5094648

[9] Hendrick AM , Gibson MV , Kulshreshtha A . Diabetic retinopathy. Prim Care. 2015;42(3):451‐464. doi:10.1016/j.pop.2015.05.005 26319349

[10] Hammes HP . Diabetic retinopathy: hyperglycaemia, oxidative stress and beyond. Diabetologia. 2018;61(1):29‐38. doi:10.1007/s00125-017-4435-8 28942458

[11] Miller WP , Sunilkumar S , Giordano JF , Toro AL , Barber AJ , Dennis MD . The stress response protein REDD1 promotes diabetes‐induced oxidative stress in the retina by Keap1‐independent Nrf2 degradation. J Biol Chem. 2020;295(21):7350‐7361. doi:10.1074/jbc.RA120.013093 32295843 PMC7247303

[12] De La Cruz JP , González‐Correa JA , Guerrero A , de la Cuesta FS . Pharmacological approach to diabetic retinopathy. Diabetes Metab Res Rev. 2004;20(2):91‐113. doi:10.1002/dmrr.432 15037985

[13] Suryavanshi SV , Barve K , Utpat SV , Kulkarni YA . Triphala churna ameliorates retinopathy in diabetic rats. Biomed Pharmacother. 2022;148:112711. doi:10.1016/j.biopha.2022.112711 35168075

[14] Laddha AP , Kulkarni YA . Daidzein ameliorates diabetic retinopathy in experimental animals. Life Sci. 2021;265:118779. doi:10.1016/j.lfs.2020.118779 33217441

[15] Kowluru RA . Effect of reinstitution of good glycemic control on retinal oxidative stress and nitrative stress in diabetic rats. Diabetes. 2003;52(3):818‐823. doi:10.2337/diabetes.52.3.818 12606525

[16] Huang L , You J , Yao Y , Xie M . High glucose induces pyroptosis of retinal microglia through NLPR3 inflammasome signaling. Arq Bras Oftalmol. 2021;84(1):67‐73. doi:10.5935/0004-2749.20210010 33470344 PMC12289150

[17] Adeva M , González‐Lucán M , Seco M , Donapetry C . Enzymes involved in l‐lactate metabolism in humans. Mitochondrion. 2013;13(6):615‐629. doi:10.1016/j.mito.2013.08.011 24029012

[18] Hsieh YS , Yeh MC , Lin YY , et al. Is the level of serum lactate dehydrogenase a potential biomarker for glucose monitoring with type 2 diabetes mellitus? Front Endocrinol. 2022;13:1099805. doi:10.3389/fendo.2022.1099805 PMC9801409 36589820

[19] Wang YL , Koh WP , Yuan JM , Pan A . Association between liver enzymes and incident type 2 diabetes in Singapore Chinese men and women. BMJ Open Diabetes Res Care. 2016;4(1):e000296. doi:10.1136/bmjdrc-2016-000296 PMC5030569 27738514

[20] Arnqvist HJ , Westerlund MC , Fredrikson M , Ludvigsson J , Nordwall M . Impact of HbA1c followed 32 years from diagnosis of type 1 diabetes on development of severe retinopathy and nephropathy: the VISS study. Diabetes Care. 2022;45(11):2675‐2682. doi:10.2337/dc22-0239 36094113

[21] Lind M , Pivodic A , Svensson AM , Ólafsdóttir AF , Wedel H , Ludvigsson J . HbA(1c) level as a risk factor for retinopathy and nephropathy in children and adults with type 1 diabetes: Swedish population based cohort study. BMJ. 2019;366:l4894. doi:10.1136/bmj.l4894 31462492 PMC6712507

[22] Yang H , Xia M , Liu Z , et al. Nomogram for prediction of diabetic retinopathy in patients with type 2 diabetes mellitus: a retrospective study. J Diabetes Complications. 2022;36(11):108313. doi:10.1016/j.jdiacomp.2022.108313 36183450

[23] Chen D , Sun X , Zhao X , Liu Y . Associations of serum uric acid and urinary albumin with the severity of diabetic retinopathy in individuals with type 2 diabetes. BMC Ophthalmol. 2020;20(1):467. doi:10.1186/s12886-020-01713-5 33256661 PMC7706232

[24] Xia Q , Zhang SH , Yang SM , et al. Serum uric acid is independently associated with diabetic nephropathy but not diabetic retinopathy in patients with type 2 diabetes mellitus. J Chin Med Assoc. 2020;83(4):350‐356. doi:10.1097/jcma.0000000000000285 32132382 PMC13047968

[25] Guo Y , Liu S , Xu H . Uric acid and diabetic retinopathy: a systematic review and meta‐analysis. Front Public Health. 2022;10:906760. doi:10.3389/fpubh.2022.906760 35712295 PMC9197488

[26] Zeng J , Chen M , Feng Q , et al. The platelet‐to‐lymphocyte ratio predicts diabetic retinopathy in type 2 diabetes mellitus. Diabetes Metab Syndr Obes. 2022;15:3617‐3626. doi:10.2147/dmso.S378284 36444389 PMC9700435

[27] Yue S , Zhang J , Wu J , Teng W , Liu L , Chen L . Use of the monocyte‐to‐lymphocyte ratio to predict diabetic retinopathy. Int J Environ Res Public Health. 2015;12(8):10009‐10019. doi:10.3390/ijerph120810009 26308022 PMC4555325

[28] Luo WJ , Zhang WF . The relationship of blood cell‐associated inflammatory indices and diabetic retinopathy: a meta‐analysis and systematic review. Int J Ophthalmol. 2019;12(2):312‐323. doi:10.18240/ijo.2019.02.20 30809490 PMC6376234

[29] Tight blood pressure control and risk of macrovascular and microvascular complications in type 2 diabetes: UKPDS 38. UK prospective diabetes study group. BMJ. 1998;317(7160):703‐713. 9732337 PMC28659

[30] Efficacy of atenolol and captopril in reducing risk of macrovascular and microvascular complications in type 2 diabetes: UKPDS 39. UK prospective diabetes study group. BMJ. 1998;317(7160):713‐720. 9732338 PMC28660

[31] Ishihara M , Yukimura Y , Aizawa T , Yamada T , Ohto K , Yoshizawa K . High blood pressure as risk factor in diabetic retinopathy development in NIDDM patients. Diabetes Care. 1987;10(1):20‐25. doi:10.2337/diacare.10.1.20 3568963

[32] Cui J , Ren JP , Chen DN , et al. Prevalence and associated factors of diabetic retinopathy in Beijing, China: a cross‐sectional study. BMJ Open. 2017;7(8):e015473. doi:10.1136/bmjopen-2016-015473 PMC5724071 28855199

[33] Ding J , Wai KL , McGeechan K , et al. Retinal vascular caliber and the development of hypertension: a meta‐analysis of individual participant data. J Hypertens. 2014;32(2):207‐215. doi:10.1097/HJH.0b013e32836586f4 24322199 PMC4120649

[34] Ikram MK , Witteman JC , Vingerling JR , Breteler MM , Hofman A , de Jong PT . Retinal vessel diameters and risk of hypertension: the Rotterdam study. Hypertension. 2006;47(2):189‐194. doi:10.1161/01.Hyp.0000199104.61945.33 16380526

[35] Sueishi K , Sumiyoshi S , Nakashima Y . Pathological characteristics of diabetic macro‐angiopathy. Nihon Rinsho. 2006;64(11):2005‐2011. 17087290

